# Distinct activities of HTLV-1 Tax1 from HTLV-2 Tax2 play key roles in pathogenesis

**DOI:** 10.1186/1742-4690-8-S2-O22

**Published:** 2011-10-03

**Authors:** Masahiro Fujii, Masahiko Takahashi, Manami Yoshita, Michitaka Imai, Masaya Higuchi

**Affiliations:** 1 Niigata University, Japan

## 

While human T cell leukemia virus type 1 (HTLV-1) is an etiological agent of adult T-cell leukemia (ATL), its close relative HTLV-2 is not associated with any leukemia. HTLV-1 and HTLV-2 encode Tax1 and Tax2 proteins, respectively, which are essential for immortalization of T-cells by the respective viruses, thereby persistent infection. Tax1 and Tax2 have more than 75% amino acid similarities, but we show here that Tax1 and Tax2 have multiple distinct activities. Tax1 induced IL-2-independent growth of a T-cell line CTLL-2 more efficiently than Tax2. By contrast, Tax2 immortalized human T-cells in the presence of IL-2 more efficiently than Tax1. These results suggest that HTLV-1 and HTLV-2 have distinct IL-2 requirements to immortalize T-cells. We also found that Tax1 altered cellular oxidative stress response. Arsenite, a pro-oxidant, induced stress granule (SG) formation, which functions as a protective role against oxidant-induced cell damages, but the formation was inhibited by Tax1. We will discuss these findings in terms of the HTLV-1-specific pathogenesis.

